# Graduate experiences with transnational nursing education: a qualitative enquiry

**DOI:** 10.1108/IJHCQA-06-2018-0155

**Published:** 2019-04-15

**Authors:** Vasanthrie Naidoo, Maureen Nokuthula Sibiya

**Affiliations:** Department of Nursing, Durban University of Technology, Durban, South Africa

**Keywords:** Cross-border nursing education, Transnational nursing education

## Abstract

**Purpose:**

The purpose of this paper is to share insights, research findings and discuss key issues related to graduate experiences with transnational nursing education (TNE).

**Design/methodology/approach:**

The authors used a qualitative approach and sampled national and international nurse graduates to identify challenges and best operating practices in cross-border nursing program facilitation.

**Findings:**

This research paper has provided a platform for graduates to lend their voices to the promotion of effective cross-border nursing education delivery and suggests that although international collaborations endeavor to maintain high academic standards in TNE, there is still a need to re-engineer, revise and adapt curricular content, learning, teaching and assessment practices to aid the nursing student.

**Research limitations/implications:**

Identified challenges affecting the facilitation and delivery of cross-border nursing education programs can act as levers to improving service quality of present and future cross-border programs to the nursing student. This will assist future nursing students to recognize culture shock and embrace their decision to pursue nursing.

**Practical implications:**

The experience of being involved in TNE for nursing students may not be that much different than students of other disciplines. While not able to be generalized to the entire population, the reports by the nursing students in this sample appear to be valuable and worthwhile to continue supporting and encouraging other TNE opportunities.

**Originality/value:**

This paper explores cross-border nursing education experiences from national and international perspectives. The authors were able to explore inherent TNE challenges from diverse population and cultural backgrounds.

## Introduction

Transnational education has become a reality in countries with inadequate education opportunities, based on the fact that education is considered a vital component for a country’s economic development, social care services and improved living standards (Fearnside and Chung, 2017). Thus, many nationals from different countries around the world are taking higher education seriously, which is consequently, translating cross-border education, into transnational nursing education (TNE) or cross-border nursing education. Knight (2006) defines cross-border nursing education or TNE as all higher education nursing programs or educational offerings in which student nurses study in a country different from the one where the awarding institution is based. Knight also argues in a separate study that these educational offerings are fundamental for meeting ever-increasing demands for nurse training around the world. The author also concludes that transnational education can provide opportunities to increase a nation’s health and welfare by ensuring adequately trained and efficient human health resources (Knight, 2008).

Our purpose was to explore nursing students’ TNE experiences and to identify benefits and impediments that can be used to shape existing and future TNE programs. Nursing education internationalization necessitates consideration regarding factors, such as friendships, cultural and religious acceptance and adaptation to new norms and traditions, but according to Hurst and Patterson (2014), factors such as recruitment, training and appropriate workforce development are also vital for successful social care delivery. Harvey *et al.* (2017) agree that for any successful TNE implementation and positive graduate experience, clear quality guidelines need to be pre-determined to avoid challenges that encompass TNE ventures.

Cross-border nursing graduate experiences have not been clearly explored nor understood in the literature. Edmonds (2010) states that although interest has increased regarding study abroad programs over the last decade, many nurse researchers recommend that further inquiry is necessary to provide support for this educational practice and address the gap in the existing nursing literature. It was, therefore, imperative that we explore cross-border nursing education programs and identify their impact on the cultural competence development and global perspectives in nursing graduates’ lives. We attempt to address this unmet need by enumerating success and persistence dynamics as these qualities manifest in TNE participants’ lives and in their personal stories.

## Purpose and problem statement

Our purpose was to explore nursing graduates transnational nursing program experiences and to identify any benefits or impediments that could be used to promote quality to any existing and future cross-border programs. Throughout the world, international students are important, thereby, increasing the global competitiveness for competent nursing graduates (Knight, 2004). Educational institutions that offer cross-border nursing programs have primarily focused on language competency and cultural diversity as entrance and completion criteria. What has been omitted in these services is recognizing the impact this had on the nursing student who has to interact with strangers, often in a strange learning/teaching environment for an extended time to undertake further education and training (Edmonds, 2010). Our reflections on our working experience with international students identified that students in such situations experience various challenges and distress levels, which affect learning outcomes and their quality of life.

## Significance

Existing nursing literature focuses on support for internationalized nursing education. Despite the many advantages and benefits attributed to cross-border nursing, current nursing literature regarding graduate experiences remains limited. By exploring such gaps, we believe that cross-border nursing graduates’ personal stories will raise issues about graduate nurses’ social experiences for university administrators, which should stimulate discussions and promote new model and framework development to support cross-border nursing program facilitation.

## Literature review

Our review was relevant to internationalized nursing education or “importing” and “exporting” nursing education. According to Altbach and Knight (2007), efforts to monitor international initiatives and ensure quality are integral to international higher education environments.

## International and national TNE facilitation

Shaffer and Dutka (2013) state that the need for global standards in nursing education has arisen for several reasons, such as the increasing complexities in healthcare provision, the increasing health professional role at different levels and the need to assure more equitable access to healthcare for the country’s citizens. Martin and Stella (2007), who explored international institutional educational planning, stated that academic preparedness, coordinating, teaching or facilitating international students may not have kept abreast with the growth in university student enrollments in transnational teaching programs. Zhou (2014) studied Chinese academia and nursing student viewpoints related to TNE and concluded that there was a need for educators to develop specific strategies to ensure positive student experiences. Zhou also revealed that this was also necessary to enhance positive facilitator or coordinator TNE teaching experience. A Malaysian study revealed that educational ideologies should be considered when teaching students from different cultural backgrounds to ensure successful TNE (Arunasalam, 2013). Seaton (2010) emphasizes cultural sensitivities associated with teaching international students and affirms the need for educators to challenge traditional teaching approaches by utilizing culturally appropriate methods to successfully attain learning/teaching outcomes. According to Moleki (2008), distance education programs are perceived as problematic due to the lack of physical or face-to-face lecturer contact. Moleki noted that the lecturer was responsible and accountable for arranging suitable communication between him/her and the student. The author, however, cautioned about creating a balance between supporting and guiding the student without creating dependency and advocated for a student learning culture that instilled self-sufficiency, self-directedness and independence.

## Student experiences with TNE

Nurses’ motives for enrolling in TNE programs were mainly to obtain a western degree, naming the financial benefits and incentives as extrinsic motivators. However, drawing on their resilience, some nurses developed self and professional independence and transformation. Most students involved in these programs also depended on clinical instructors and book knowledge as authoritative sources. Miliszewska (2008) stated that students verbalized that they became critical readers and writers during the teacher’s or lecturer’s absence. However, Arunasalam (2013) argued that cross-border nursing education students reiterated that being only taught theory without application was not appreciated as they were unable to link theory to practice. Miliszewska also revealed that students complained that throughout the program they had to undertake online sessions to complete their assessments for achievement purposes. Miliszewska concluded that the theory–practice gap evident in their clinical nursing practice resulted in nursing students being unable to achieve their learning outcomes and manage the cross-cultural diversity in nursing. This further highlighted the need for cultural sensitivity and awareness in cross-border nursing education.

## Methodology

### Research population

An interpretive research paradigm was used together with a qualitative, multiple case-study approach to identify challenges and best operating practices from cross-border nursing graduates’ viewpoints. Nursing graduates involved in nurse training were considered to be TNE direct recipients. Therefore, this sample comprised a purposive sampling strategy, whereby nursing graduates (both local and international), who had undergone nurse education and training, were invited to participate. Data were gathered via Skype interviews.

### The graduate’s position within the institution

Students fitting graduate descriptions and ones who had participated in a TNE program were sought within the educational system, because of their potential impact on TNE policy decision making and implementation. Additional participants were considered if they had any responsibility or accountability in any off-shore program within a participating institution.

### The graduate’s institutional understanding

Those graduates who were thought to be able to best meet the study objectives were selected in consultation with educational institution staff and interviewed accordingly. Graduates were then interviewed until data saturation was reached. Data saturation in research studies occurs when the researcher samples to the point where no new information is obtained, and redundancy is achieved (Polit and Beck, 2012).

### The graduate’s knowledge related to governance structures

It was vital that interviewee graduates understood educational system governance related to nursing practice and education, which allowed the researcher to gain situational insight, understanding and meaning in addressing graduates’ individual experiences. Our literature search had identified global challenges related to TNE facilitation. Thus, the impetus was to sample national and international participants as this assisted in drawing a comparison between national and international TNE practices. The sample comprised national and international nursing graduates (Table I). Probing type questions were used to guide the interviews, during which participants articulated their responses by giving an in-depth account and narrated their experiences surrounding their participation in TNE:

1. What are your current roles and responsibilities in your nursing profession?
2. When were you involved with cross-border or TNE?
3. Were you orientated to the program?
4. Did the program meet with your expectations? Please motivate your answer.
5. What were your thoughts on the recruitment and selection processes for the program?
6. What measures were in place to assist and support students from the foreign country?
7. Was there any feedback or evaluation from students to the program coordinator?
8. How do you think a program of this nature supports your professional development?
9. What challenges did you encounter during this type of teaching/learning or administration of the program?
10. What were some of the challenges that were brought to the coordinators’ attention and what was done about it?
11. What would you consider to be beneficial about the program and how did this affect you?
12. What would you recommend as additional factors or best operating practices to assist and support staff and students engaging in TNE?

### Setting

Selecting a suitable setting is a vital component for effective data collection in a research study. According to Grove *et al.* (2013), qualitative studies conducted in a natural setting means that the researcher cannot manipulate or change the study environment that allows a rich process, people and interaction mix, which assists in addressing the study’s research questions. We selected participants who had been involved in TNE after permission was sought, granted and approved.

### Data collection tool pre-test

Meyer *et al.* (2009) define a pre-test as one that is conducted to test, validate and refine data collection instruments. In our study, a pre-test study was conducted with five graduates who had been involved in TNE. These participants were not included in the main study.

### Interviews

Interviews are known to capture interviewees’ unique experiences and special stories and produce data as words Grove *et al.* (2013). Autonomy was maintained by obtaining informed consent from participants. Semi-structured interviews were conducted, and most questions were open-ended and designed to address our research questions. Interview questions focused on organizational structures and core processes related to cross-border nursing education facilitation such as curriculum development, faculty evaluation and development and student learning and teaching. Interviews were scheduled for 30–35 min and audiotaped, which helped to provide unobtrusive and accurate recordings. Total interviews were guided by data saturation. All interviews were later transcribed by the researcher with the participants’ permission.

## Ethical considerations

Ethical clearance was obtained from the University Institutional Research Ethics Committee and a written consent was obtained from all participants, who made an informed, voluntary decision to participate in the study.

## Trustworthiness

The study’s qualitative nature allowed Lincoln and Guba’s (1985) strategy (credibility, transferability, dependability and confirmability) to be applied to enhancing trustworthiness. Credibility in this study was achieved by accurately describing the study’s parameters such as whom, where and when (Polit and Beck, 2012). Transferability was promoted by ensuring that the research process was accurately described to all participants. Descriptions regarding data gathering, data analysis and interpretation attained dependability, while voice recordings and field notes increased confirmability.

## Findings

### Graduate responses

#### Theme 1: the distance factor between teacher and student

Our findings revealed that despite having a cordial relationship with the educator, the increased physical distance between educator and student was often a major problem in the student achieving his or her learning outcomes. Students felt that with modern technology, lecturer accessibility will not be an issue. However, having online sessions and tutorials did not replace the one-on-one contact between student and lecturer. It was also thought that the relationship between graduate and lecturer was to a certain degree affected by distance. Learning opportunities for spontaneous interactions between facilitator and student using the “teachable moment” was indeed hindered and limited:

I had a lot of obstacles because I never had personal guidance from my lecturer […] your lecturer guides and motivates you

(Graduate 3).

Participants also felt that the lecturer’s presence would help overcome any resistance or challenges they could have faced, when the theory was practiced in the clinical setting as they would have benefited from the guidance, advice and support from their lecturer at that moment. Other interviewees stated that their instructional learning was strengthened with online media such as Skype, YouTube and Facebook, which enabled query and complaint handling:

As students, we were very happy to see our lecturer from time to time […] it gave us a chance to clarify all our doubts

(Graduate 5).

Some participants felt “all alone.” They stated that when their educator was around, they felt safe. As soon as she left them, they felt uncertain and unsure how they should respond to situations that faced them in the clinical settings:

Nobody could relate to us like our teacher […] she was our mentor and she was our friend […] when she was away […] it was like nobody understood what you did […] you were just left alone.

(Graduate 9).

#### Theme 2: learning opportunities

Interviewees pointed out that TNE learning opportunities were student centered, which meant that although there were common nursing subjects or common subject content, in which all students needed to learn to pass, each student approached the subjects from their own perspectives, experience and understanding. Each student embarked on the course, having their own specific learning needs and expectations. Graduates responded that their experiences were, therefore, unique to them personally and valued their own personal learning journey. Some graduates stated that other structured learning support, such as computer skills and language skills, was designed to help their learning development:

[…] At the beginning of the course, I was confused and overwhelmed […]. Now with bedside teaching support and clinical facilitation, I have grown in my profession and became a stronger person

(Graduate 6).

I was very nervous and did not know what to expect from the course or the instructors. I was literally terrified after the orientation […] but with the scheduled learning opportunities like clinical accompaniment, I overcame my fears and have grown professionally and have developed confidence in performing my duties

(Graduate 10).

Our findings revealed that peer mentoring was a vital strategy in helping nursing students to attain their learning outcomes. Peer mentoring was, however, not consistent in all institutions and for some nursing students, placement in the clinical environment proved frustrating as they were not given support and supervision. Students came from different countries, academic backgrounds, cultures and teaching and learning traditions, so it was a challenge to ensure sufficient teaching and learning opportunities to understand the content that was being taught. Formal lectures forming the main class interaction appeared to be the commonest way to facilitate the course:

[…] I was very comfortable with peer mentoring, it really assisted me with issues I could not understand […] even with the integration of textbook knowledge

(Graduate 2).

[…] the peer mentors actually help when our teachers are not around […] and with their guidance we gradually became competent […] and sometimes experience is the best teacher […]. because they were once in our shoes

(Graduate 9).

#### Theme 3: support structures

Graduates verbalized a positive opinion on the support that was given during their studies. They also felt that this encouraged them to embark on future study abroad opportunities. Some stated that the support they gained from their lecturers and facilitators was invaluable and it was only their dedicated efforts that they felt their successes had increased:

I now realize the importance of having an international curriculum for my employability […] but it would not have been possible were it not for our program facilitator […] there were times I wanted to quit […] she basically held our heads above water

(Graduate 3).

Some students complimented their TNE coordinators for understanding multiculturalism’s inherent values as it provided support when they faced barriers to learning such as language deficits. Other students noted with appreciation the support they received from the educational institution with legalities and “red tape” issues. The following response highlights this:

[…] bribery and corruption prevented us from getting our academic records from our previous institution […] eventually our facilitator had to contact the Heads of Department and send through endorsements which hastened the process

(Graduate 9).

Some graduates said that they were not properly orientated to the program. Others stated that important issues like learning material, transportation and accreditation were sometimes discussed in a “by the way” fashion. It was difficult for most students to understand TNE governance in their country. Some graduates felt that although the nursing programs were at times offered simultaneously in two different continents, different educational systems created problems with accreditation and recognition causing frustration and delays:

There should be a general legal framework, which could integrate the different educational structures and values of the two countries in issuing the same qualifications […] after all we are doing the same course

(Graduate 10).

[…] like being told everything on orientation […] whether we are going to get jobs when we go to another country or whether we have to write another exam […] we have a right to know this as we pay a lot of money for the courses

(Graduate 5).

#### Theme 4: group cohesion

All participants stated that they valued the support they received from their fellow students. They also stated that the home-like environment at times increased group morale and added to their confidence and abilities. According to Sahoo and Ghosh (2016), physical settings such as ambience, layout, facilities and healthcare service infrastructure, play an important role in influencing the satisfaction in any work environment. Participants stated that working in groups in and outside the classroom fostered a family-like feeling that helped them overcome loneliness that they felt being away from their loved ones. Their cultural oneness allowed them to practice their traditions and remain connected. Stronger academic students would assist by explaining course content in their home language to overcome language barriers to learning. Graduates stated that friendships and relationships forged still remained strong nowadays despite being across continents. Graduates welcomed different and interesting approaches to research-based learning in a transnational context but found it useful to have others with research experience sharing their research knowledge with them and finding ways to include each other in research activities. Participants also stated that having the support to develop the skills to carry out their research encouraged them in higher degree studies:

I learnt so much about research from my batch mate […] that I can now carry out research in my own teaching and learning

(Graduate 5).

[…] we were from different states but grew close […] my friend was my pillar of strength […] she shared my anxieties, awkward situations, frustrations, moments of confusion and doubt

(Graduate 1).

## Discussion

### Theme 1: the distance factor between teacher and student

TNE effectiveness is directly related to the extent to which the facilitator is able to empower students and mentors to meet clinical outcomes. In this theme, the lecturer and student relationship was challenged by physical distance, which resulted in the student becoming demotivated owing to physically being a long distance away from the facilitator. Our findings revealed that the physical distance between facilitator and the student was a major problem, as online sessions were not the same as face-to-face contact. Opportunities for spontaneous interaction with the teacher or facilitator were limited. Despite having comprehensive tutorial materials, which were designed to assist students to achieve his or her outcomes, gaps persisted. Teimourtash *et al.* (2014) state that with the increased distance in TNE, communication bridging should be typically facilitated with online instructional learning such as podcasts, e-mail and video or telephone conferencing.

In the weaker student, the increased physical distance between educator and student can become a challenge and can leave the student less confident, less self-directed and with a desire to spend more time with the lecturer. Arunasalam (2013), therefore, suggests that the emotional maturity level varies from individual to individual and is based on TNE expectations or past experiences. Mendes and de Jesus Jose Gil Fradique (2014) agree that those who lead organizational processes, such as coordinators and facilitators, have the responsibility for promoting a climate that will enhance the nurse’s performance, thereby favoring the nurses’ professional development.

Madakane (2011) agreed that in most transnational universities, geographical separation, size differences and structures can pose difficulties, but structures should be in place to allow those involved in transnational programs to actively work to achieve a student’s sense of belonging and staff alike. Daniel *et al.* (2005) noted that management systems that support e-Learning were effectively utilized to bridge the distance gap between learner and educator. The same study revealed that teachers and institutions around the world created and shared instructional learning materials and courses for use on cross-border platforms. Online learning material on cross-border platforms can provide a conduit between teacher and student.

### Theme 2: learning opportunities

In nursing, theory and practice are married and involve lifelong learning. Theory–practice integration is considered inseparable in nursing and the knowledge gained from nursing education adds value and benefit to the students’ clinical abilities. Lifelong learning in nursing relates directly to quality care and gaining relevant knowledge and skills (Searle *et al.*, 2009). Cross-border teaching needs to carefully consider learning and teaching needs to ensure high-quality teaching and support. With TNE, preceptors and mentors are identified as key persons regarding learning opportunities as they are experienced clinicians who provide individual guidance to a less experienced nurse. The main objective in assigning students to the preceptor in clinical practice is to bridge the gap between workplace reality and academic environment idealism or putting it simply – to integrate theory and practice. Insufficient mentorship or preceptorship, in the educator’s absence, is another challenge for TNE, and often student supervision and teaching during clinical placement sometimes does not align to program requirements. Moleki (2008) states that knowledge application requires expert decision making, clinical problem solving and effective reasoning skills, which are fundamental in nursing education, and opportunities for theory–practice integration can be enhanced by using the available learning opportunities.

Perrin (2015) looks at knowledge in distance learning as a network and considers learning to be a networking process utilizing people, books, websites, programs and databases connected by internet, intranet or direct contact. Perrin adds that any TNE student should readily engage in online networking. An American study used simulation and case-based learning as innovative learning opportunities to examine the differences in learning and teaching strategies in student nurse performance, revealing that nurses were confident and displayed achievement in all nursing competencies (Nicholson, 2010).

### Theme 3: support structures

While TNE programs include diversified, race-groups and cultures, responses from participants clearly demonstrated the differences in individual coping strategies and these often related to adjustments and adaptations when faced with different situations in new environments with new people. According to Arunasalam (2013), adjustment and adaptation require a conscious learning process as initial emotions and students’ thoughts on learning from foreign nationals or learning in a foreign place can generate responses that usually result in “flight or fight” reaction. Arunasalam adds that although people may adjust quickly over time, these differences allow the student to become accustomed to multiculturalism, while they embrace new opportunities and knowledge.

Our findings revealed that common issues troubling graduates were related to transportation and miscommunication. O’Neill and Chapman (2015) shows similarities with masters’ degree students engaging in cross-border studies. Challenges experienced by them were related to language barriers to learning, housing and logistics. According to the CHE’s (2015) annual report, specific criteria were laid down for program accreditation. It was also noted that cross-border programs needed to be governed by policies that guided resources and support services, such as library and information technology, both on and off campus. The main reasons adequate structure and support mechanisms are necessary are to ensure that students and foreign partner institutions are protected from low-quality service providers and that they have a relevant and high-quality educational experience. These criteria were in keeping with participants’ responses in this study, as loop-holes in the TNE program were attributed to legalities and accreditation issues surrounding the program delivery.

Middlehurst and Woodfield (2004) in the UK discussed TNE program effects on students and found that many who experienced difficulties with time management, owing to new foreign learning and teaching cultures, had to attend individual student counseling sessions to learn to manage their time more effectively. The same authors concluded that other support structures, like having an English and communication module embedded into TNE curriculum, overcame language barriers to learning.

### Theme 4: group cohesion

Although graduates in our study were skilled and knowledgeable, it was fair from their responses to assume that a positive climate between fellow students allowed a positive and creative learning climate. Burnett (2008) revealed that job satisfaction can be attributed to group cohesion and can be derived from feelings and emotions perceived by the individual employee based on work experiences. These positive environments were excellent for gaining experience and building student confidence and competence. Lephalala (2006) agreed that students stayed in their workplaces or study groups owing to positive peer influence. Teimourtash *et al.* (2014) urged readers to consider TNE as an “opportunity” rather than a “deficit” model, as distance learners bring experience, abilities, resources and learning encounters to each other that can only serve to enrich learning. Madakane (2011) explored student satisfaction; findings indicated that student satisfaction is influenced by program quality or communication between lecturers or facilitators and by supportive learning environment. The study revealed that relationships between students may range from strictly cultural to a highly relational bond. The author further stated that when students engaged in a relationship, they bonded with each other, resulting in a unified disposition toward a desired goal.

### This study’s contribution to knowledge

Cross-border nursing education appears to be the way forward in this century. The findings and lessons we learnt signify the increased personal growth, cultural diversity awareness, adaptation to an unfamiliar environment and increased cross-border students’ self-efficacy. These findings are also relevant to all graduates engaged in international partnerships related to non-nursing cross-border programs and expands stakeholder knowledge, ensuring quality in TNE teaching and learning. Further areas for research based on the findings are to explore clinical facilitator and mentor experiences with cross-border nursing students and clinical accompaniment to ascertain further challenges from a different perspective. Our findings suggest more research to be done to determine whether partnering institutions have implemented best operating practices, such as quality assurance, to govern cross-border programs.

## Conclusion

According to Edmonds (2010), any student who has the unique opportunity to study “out of their comfort zone” will create a memorable opportunity to learn how to adapt, which will assist future nursing students to recognize culture shock and embrace their decision to pursue nursing. The TNE experience and involvement for nursing students may not be that much different from other students. While we are not able to be generalized to the entire population, the nursing students in this sample appear to be valuable and worthwhile to continue supporting and encouraging other TNE opportunities. Quality nursing education delivery will always remain a main focus in any tertiary institution, where there are possible risks related to inappropriate teaching and learning programs in off-shore environments. This research provides a platform for graduates to lend their voices to promoting cross-border nursing education delivery and suggests that although international collaborations endeavor to maintain high academic standards in TNE, there is still a need to re-engineer, revise and adapt curricular content, learning and teaching and assessment practices to aid nursing students. It is, therefore, hoped that our findings offer guidance to forthcoming and existing higher education curriculum developers who plan to engage in national or international educational partnerships.

## Figures and Tables

**Table I:** TNE graduates’ demographic profile

Participant	Position	Program	Origin country	Host country
No. 1	Registered nurse	Masters in nursing science	India	Canada
No. 2	Registered nurse	Masters in nursing science	Asia	Saudi Arabia
No. 3	Registered nurse	Critical care nursing	Asia	Saudi Arabia
No. 4	Registered nurse	Critical care nursing	Asia	Australia
No. 5	Registered nurse	Critical care nursing	India	Australia
No. 6	Registered nurse	Operating theatre nursing	India	Canada
No. 7	Registered nurse	Operating theatre nursing	Asia	UK
No. 8	Registered nurse	Operating theatre nursing	Asia	Canada
No. 9	Registered nurse	Critical care nursing	Seychelles	Kenya
No. 10	Registered nurse	Critical care nursing	Seychelles	Kenya
